# Integrated metabolomics and transcriptome analysis on flavonoid biosynthesis in safflower (*Carthamus tinctorius* L.) under MeJA treatment

**DOI:** 10.1186/s12870-020-02554-6

**Published:** 2020-07-29

**Authors:** Jiang Chen, Jie Wang, Rui Wang, Bin Xian, Chaoxiang Ren, Qianqian Liu, Qinghua Wu, Jin Pei

**Affiliations:** 1 State Key Laboratory of Characteristic Chinese Medicine Resources in Southwest China, Chengdu University of Traditional Chinese Medicine, Chengdu, 611137 China; 2 College of Pharmacy, Chengdu University of Traditional Chinese Medicine, Chengdu, 611137 China

**Keywords:** Flavonoid biosynthesis, MeJA treatment, Metabolomics, Transcriptome, Molecular mechanism, HSYA, Safflower

## Abstract

**Background:**

Safflower (*Carthamus tinctorius* L.) is an important cash crop, of which the dried tube flower is not only an important raw material for dyes and cosmetics but also an important herb widely used in traditional Chinese medicine. The pigment and bioactive compounds are composed of flavonoids (mainly quinone chalcones), and studies have reported that MeJA can promote the biosynthesis of quinone chalcones, but the mechanism underlying the effect of MeJA in safflower remains unclear. Here, we attempt to use metabolomics and transcriptome technologies to analyse the molecular mechanism of flavonoid biosynthesis under MeJA treatment in safflower.

**Results:**

Based on a UHPLC-ESI-MS/MS detection platform and a self-built database (including hydroxysafflor yellow A, HSYA), a total of 209 flavonoid metabolites were detected, and 35 metabolites were significantly different after treatment with MeJA. Among them, 24 metabolites were upregulated upon MeJA treatment, especially HSYA. Eleven metabolites were downregulated after MeJA treatment. Integrated metabolomics and transcriptome analysis showed that MeJA might upregulate the expression of upstream genes in the flavonoid biosynthesis pathway (such as *CHSs*, *CHIs* and *HCTs*) and downregulate the expression of downstream genes (such as *F3Ms*, *ANRs* and *ANSs*), thus promoting the biosynthesis of quinone chalcones, such as HSYA. The transcription expressions of these genes were validated by real-time PCR. In addition, the promoters of two genes (*CtCHI* and *CtHCT*) that were significantly upregulated under MeJA treatment were cloned and analysed. 7 and 3 MeJA response elements were found in the promoters, respectively.

**Conclusions:**

MeJA might upregulate the expression of the upstream genes in the flavonoid biosynthesis pathway and downregulate the expression of the downstream genes, thus promoting the biosynthesis of quinone chalcones. Our results provide insights and basic data for the molecular mechanism analysis of flavonoid synthesis in safflower under MeJA treatment.

## Background

Safflower, *Carthamus tinctorius* L., is a member of the Asteraceae family and is an important economic plant worldwide. Its dried tubular flowers are an important raw material for dyes and cosmetics and are also an important herb widely used in traditional Chinese medicine. As a traditional Chinese medicine, the dried tubular flowers of safflower have been widely used to improve cerebral blood flow and to treat coronary heart disease, hypertension, and cerebrovascular diseases [1, 2]. Flavonoids, particularly quinone chalcones, are associated with these therapeutic effects. Among them, HSYA is the primary active component. It has the antioxidant activities and the myocardial and cerebral protective effects [3–5].

The flavonoid biosynthesis pathway is well understood, especially in some model plants, such as *Arabidopsis* [6, 7]. Chalcone synthase (CHS) catalyzes the reaction of one molecule of 4-coumaroyl-CoA and three molecules of malonyl-CoA into naringenin chalcone which is required for the flavonoids biosynthesis. It can be hydroxylated or glycosylated to the production of carthamone or HSYA. Chalcone isomerase (CHI) can convert naringenin chalcone into naringenin. In safflower, naringenin can be hydroxylated at the 6 position and glycosylated at the 6 and 7 positions to produce 5,6,7,4′-tetrahydroxyflavanone-6,7-di-O-β-D-glucoside, or it can be hydroxylated at the 6 position and glycosylated at the 5 position to the production of 5,6,7,4′-tetrahydroxyflavanone-5-O-β-D-glucoside (neocarthamin). Additionally, flavanone 3-hydroxylase (F3H) can convert naringenin into dihydrokaempferol. It can be subsequently converted into kaempferol, and kaempferol can then be converted into quercetin [8]. With an emphasis on safflower, the research on flavonoid synthesis has gradually increased. To date, many flavonoid biosynthesis genes have been cloned in safflower, such as chalcone isomerase genes (*CHIs*) [9], chalcone synthase genes (*CHSs*) [10, 11], flavanone 3-hydroxylase genes (*F3Hs*) [12], UDP-glucuronosyltransferases genes (*UGTs*) [13], and shikimate/quinate hydroxycinnamoyltransferase genes (*HCTs*) [14].

As a well-known exogenous inducing factor, methyl jasmonate (MeJA) participates in many plant processes, ranging from plant defence to growth and development [15]. MeJA is of particular interest in plant cell engineering for producing bioactive compounds [16, 17]. It has been reported that flavonoids (mainly quinone chalcones) in safflower can be stimulated under MeJA treatment [13, 18]. However, the molecular mechanism is largely unknown.

Recently, the application of metabolomics to medicinal plants has significantly facilitated the identification of the metabolic pathways of active medicinal compounds in plants. The UHPLC-ESI-MS/MS-based, widely targeted metabolomics method has become very popular in the field of analysis and identification of plant metabolites due to the advantages of high throughput, fast separation, high sensitivity, and wide coverage. Our methodology was based on a multiple reaction monitoring (MRM) approach [19], with a self-built compounds database according to the results of Chen et al. [20]. This approach has been widely applied in plant metabolite analysis in many plants, such as rice [20], tomato [21, 22], maize [23]. Furthermore, the integration of transcriptomics and metabolomics has larger advantages in revealing the biosynthetic mechanisms of key metabolic pathways [24–26]. Therefore, it is feasible to analyse the flavonoid biosynthesis pathway under MeJA treatment by the two technologies.

Here, metabolic profiling and differential flavonoid metabolites were screened based on a UHPLC-ESI-MS/MS detection platform and a self-built database (including HSYA). In addition, transcriptome sequencing and differential transcripts were analysed. Integrated metabolomics and transcriptome sequencing was analysed based on the KEGG pathway, and the expression of different flavonoid biosynthesis genes with or without MeJA treatment were analysed by real-time PCR. The promoters of genes that were significantly upregulated under MeJA treatment were cloned and analysed. Our results provide insights and basic data for the regulation mechanism analysis of flavonoid synthesis under MeJA treatment.

## Results

### Metabolic profiling and differential flavonoid metabolite analysis

The flavonoid metabolites in safflower with and without MeJA treatment were investigated based on UHPLC-ESI-MS/MS and a self-built database (including HSYA). A total of 209 flavonoid metabolites were detected, including 62 flavones, 42 flavone C-glycosides, 40 flavonols, 20 flavanones, 18 anthocyanins, 11 isoflavones, 12 flavanols, 2 flavonolignans, 1 quinone chalcone, and 1 alkaloid. (Supplementary Table 1).

**Fig. 1:**
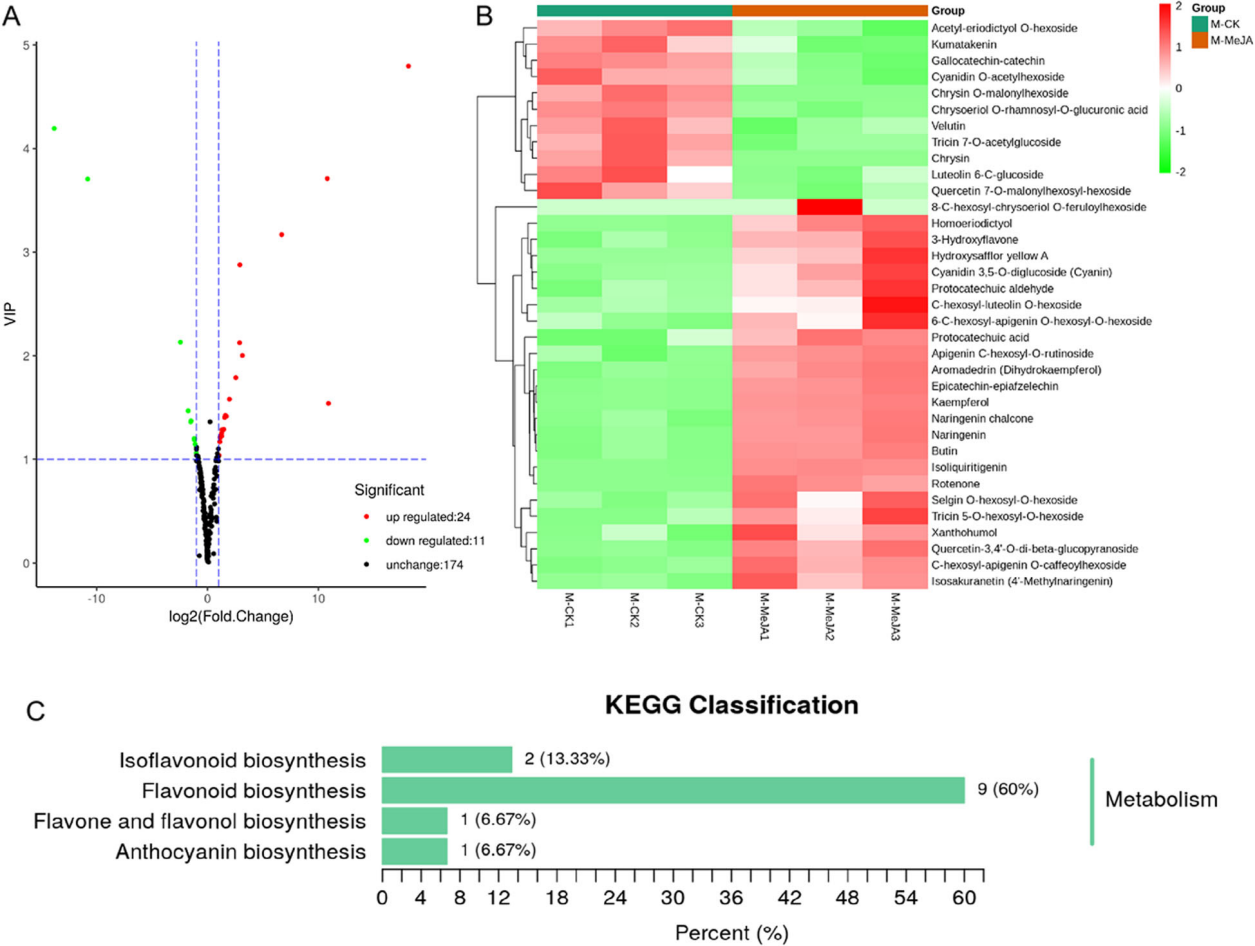
Differential metabolites analysis. **a** Volcano plot of differential metabolites. Each point in the volcanic plot represents a metabolite, the abscissa represents the logarithm of the quantitative difference multiples of a metabolite in two samples, and the ordinate represents the variable importance in project (VIP) value. The green dots in the figure represent down-regulated differentially expressed metabolites, the red dots represent up-regulated differentially expressed metabolites, and the black dots represent metabolites detected but that are not significantly different. **b** The cluster heat map for metabolites. Three biological repeats were shown in the figure. M-CK represents the flowers of safflower treated without MeJA, M-MeJA represents the flowers of safflower treated with MeJA. **c** The differential metabolites KEGG classification of the comparison flowers of safflower treated without MeJA. The proportion and number of metabolites are marked in the figure

Orthogonal partial least squares-discriminant analysis (OPLS-DA) was used for variables with less correlation. As the experiment had biological duplication, the fold change and VIP value of the OPLS-DA model were combined to screen differential metabolites. There were 35 significantly different flavonoid metabolites between MeJA-treated and untreated materials. Among them, 24 metabolites were upregulated upon MeJA treatment, especially hydroxysafflor yellow A (HSYA). Eleven metabolites were downregulated after MeJA treatment. (Fig. 1a,b) The details of the different flavonoid metabolites were provided in Supplementary Table 2. The differential flavonoid metabolites from each comparison group were screened by using the KEGG database. The KEGG classification and enrichment analysis indicated that the differential flavonoid metabolites were mainly involved in the pathway of flavonoid biosynthesis (ko00941). (Fig. 1c).

### Transcriptome sequencing and differential transcript analysis

**Fig. 2:**
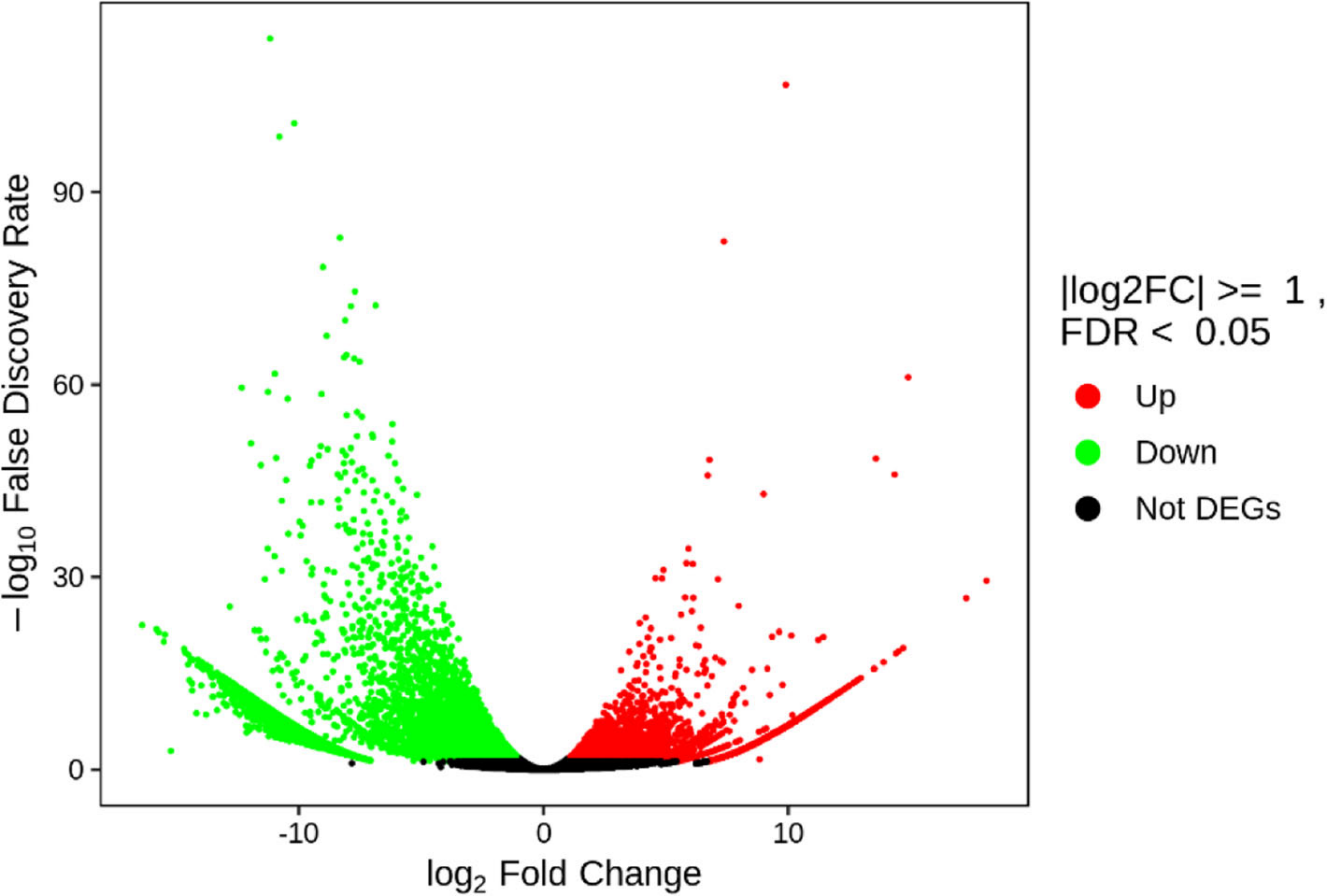
Volcano plot of differential expression genes. The abscissa represents the change of gene expression multiple (log_2_ Fold Change), and the ordinate represents the significant level of differentially expressed genes (−log10 False Discovery Rate). The expression of red gene was up-regulated, that of green gene was down-regulated, and that of black gene was not significantly different

The transcriptomes of the mixed safflower samples were sequenced. In the untreated samples,a 6.50 G clean base was obtained (44,185,480 raw reads and 43,921,238 clean reads), and Q30 was 92.40%. In the MeJA-treated samples, a 6.17G clean base was obtained (42,087,290 raw reads and 41,720,790 clean reads), and Q30 was 91.89%. The different expressed genes were analysed with DESeq2. The total number of differentially expressed genes was 31,822 (20,741 upregulated genes and 11,081 downregulated genes). (Fig. 2).

The differential flavonoid metabolite transcripts from each comparison group were screened by using the KEGG database. The experiments focused on annotations in the phenylpropane metabolic pathway, including flavonoid biosynthesis (ko00941), anthocyanin biosynthesis (ko00942), isoflavonoid biosynthesis (ko00943), and flavone and flavonol biosynthesis (ko00944). The results showed that there were 61 significantly different flavonoid biosynthesis transcripts between MeJA-treated and untreated materials (43 in ko00941, 8 in ko00942, 5 in ko00943, and 5 ko00944). Most of transcripts were involved in flavonoid biosynthesis (ko00941). The details could be viewed in Supplementary Table S3.

### Integrated analysis of the transcriptome and metabolome on the KEGG pathway

**Fig. 3:**
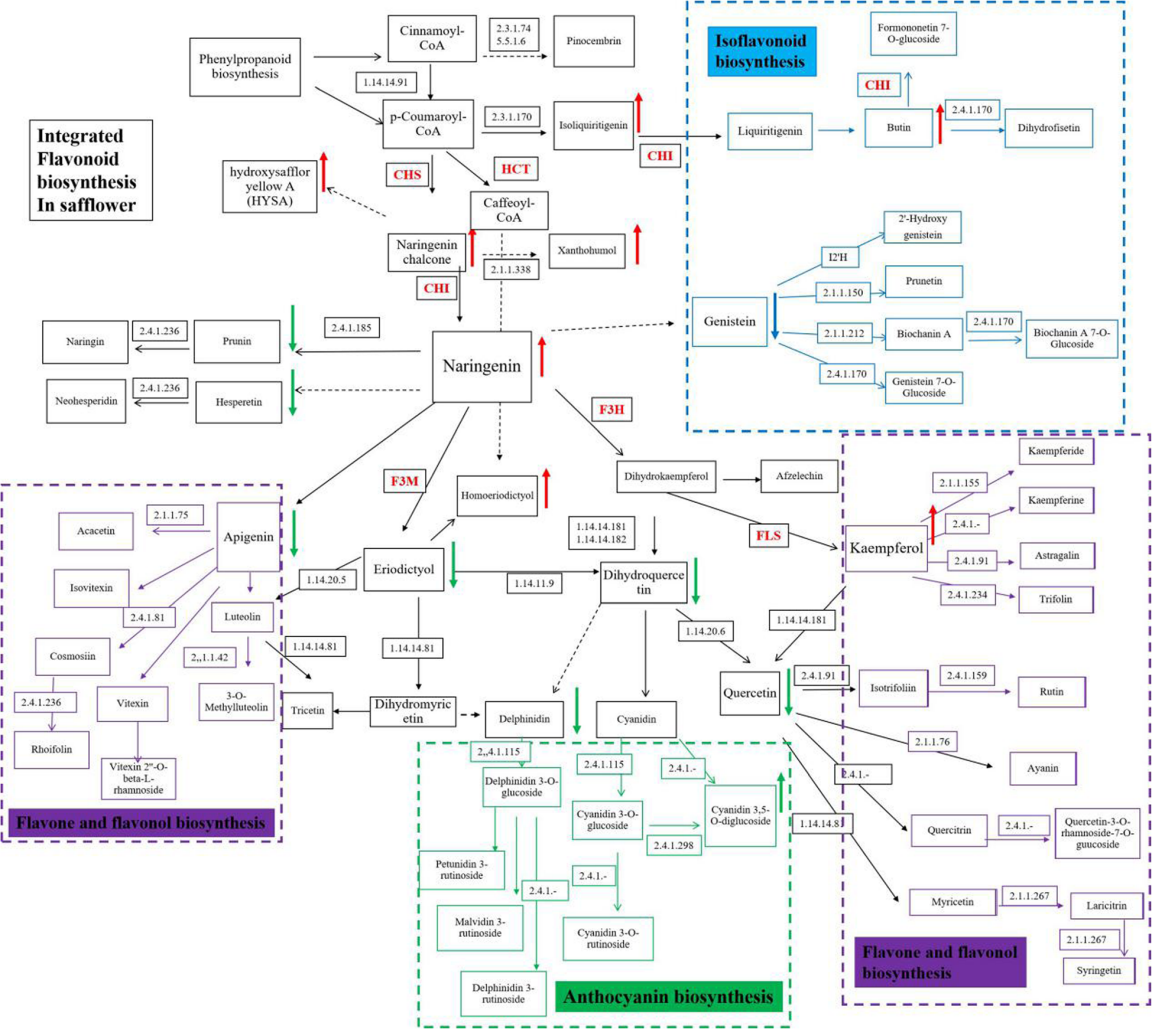
The metabolic pathway map of integrative flavonoid biosynthesis in safflower. The metabolic components were mapped into the pathway of flavonoid metabolism (including flavonoid biosynthesis (ko00941); Anthocyanin biosynthesis (ko00942); Isoflavonoid biosynthesis (ko00943); Flavone and flavonol biosynthesis (ko00944). The up arrow indicates an increase in content and the down arrow indicates a decrease in content. The reported cloned genes in safflower were tagged in the map

The metabolic components were mapped onto the pathway of flavonoid metabolism (including ko00941, ko00942, ko00943 and ko00944) by combining metabolic components that had been detected by UHPLC-ESI-MS/MS, thus constructing the metabolic pathway map of integrative flavonoid biosynthesis in safflower. (Fig. 3) At the same time, the differentially annotated metabolites, together with the differentially annotated genes, were indicated on the integrated metabolic map. (Fig. 3) From the integrated metabolic map, it was shown that MeJA might upregulate the expression of the upstream genes in the flavonoid biosynthesis pathway (such as *CHSs*, *CHIs*, and *HCTs*) and might downregulate the expression of downstream genes (such as *F3Ms*, *ANRs*, and *ANSs*), thus promoting the biosynthesis of quinone chalcones, such as HSYA.

### Expression analysis of differentially expressed flavonoid biosynthesis genes by real-time PCR

**Fig. 4:**
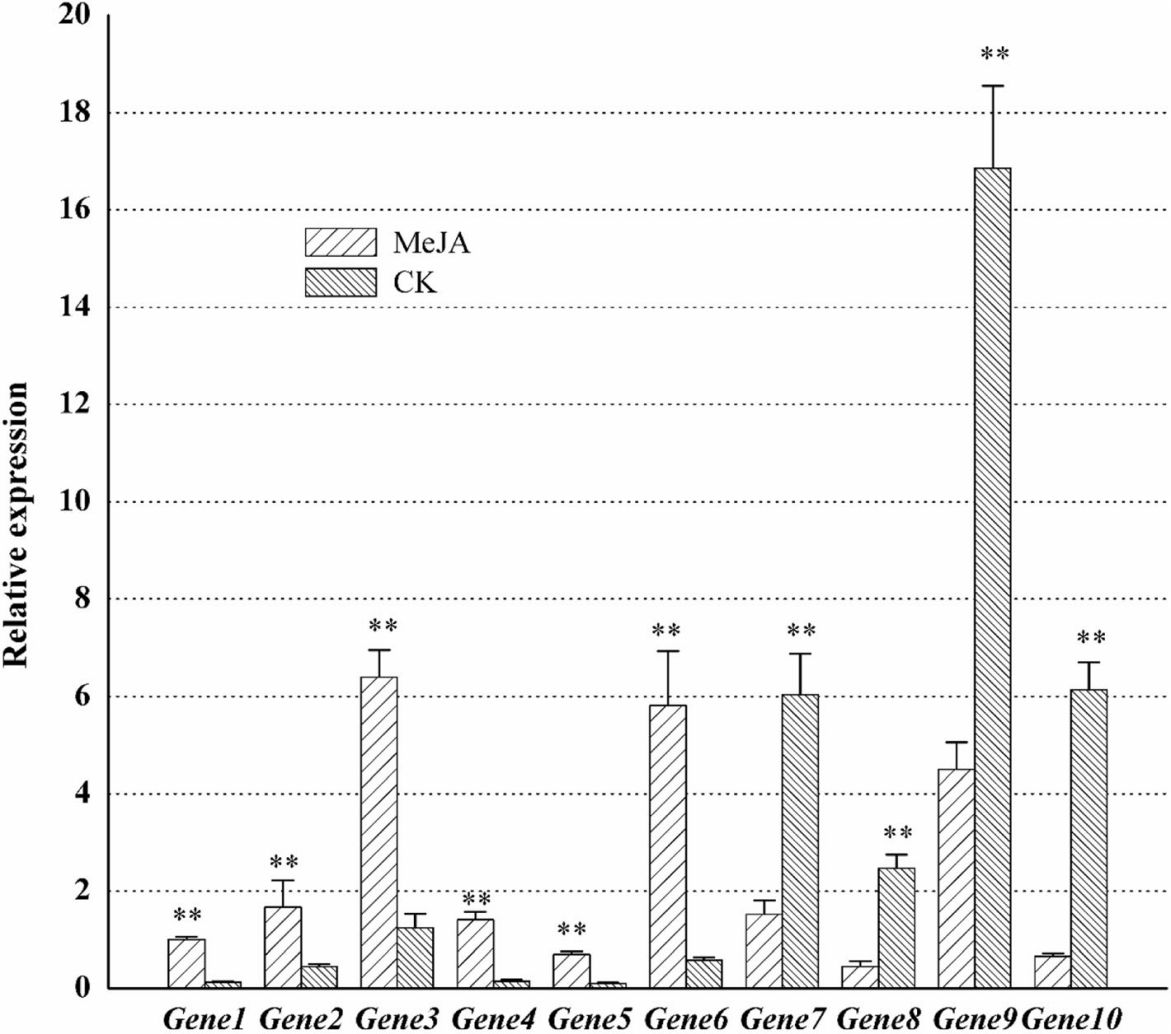
Real-time PCR expression of 10 genes from the flavonoid biosynthesis pathway. 6 genes significantly upregulated and 4 genes significantly down-regulated are included. Gene1 represents *TRINITY_DN28401_c0_g1* (annotated as *CHI)*, Gene2 represents *TRINITY_DN36537_c1_g1* (annotated as *HCT*), Gene3 represents *TRINITY_DN37574_c1_g2* (annotated as *HCT*), Gene4 represents *TRINITY_DN39063_c0_g1* (annotated as *FLS*), Gene5 represents *TRINITY_DN43344_c1_g1* (annotated as *CHI*), Gene6 represents *TRINITY_DN41734_c0_g5* (annotated as *CHS*), Gene7 represents *TRINITY_DN42700_c3_g2* (annotated as *F3H*), Gene8 represents *TRINITY_DN43120_c5_g1* (annotated as *ANR*), Gene9 represents *TRINITY_DN44094_c0_g1* (annotated as *ANS*), Gene10 represents *TRINITY_DN44565_c1_g1* (annotated as *F3M*)

Real-time PCR was used to validate the transcriptome data. Ten differentially expressed genes were selected, including *TRINITY_DN28401_c0_g1* (annotated as *CHI*), *TRINITY_DN36537_c1_g1* (annotated as *HCT*), *TRINITY_DN37574_c1_g2* (annotated as *HCT*), *TRINITY_DN39063_c0_g1* (annotated as *FLS*), *TRINITY_DN43344_c1_g1* (annotated as *CHI*), *TRINITY_DN41734_c0_g5* (annotated as *CHS*), *TRINITY_DN42700_c3_g2* (annotated as *F3H*), *TRINITY_DN43120_c5_g1* (annotated as *ANR*), *TRINITY_DN44094_c0_g1* (annotated as *ANS*), *TRINITY_DN44565_c1_g1* (annotated as *F3M*). The primers used for this experiment could be found in Supplementary Table 4. Among them, HCT contains several transferase enzymes, which include anthranilate N-hydroxycinnamoyl/benzoyltransferase that catalyses the first committed reaction of phytoalexin biosynthesis. F3H can catalyse synthesis N-terminal flavanone 3-hydroxylase. F3M can catalyse flavonoid, NADPH, H^+^, and O_2_ into 3′-hydroxyflavonoid and NADP^+^, and H_2_O, which functions like F3H. FLS catalyzes the formation of flavonols from dihydroflavonols. CHS catalyses the reaction of one molecule of 4-coumaroyl-CoA and three molecules of malonyl-CoA to form tetrahydroxychalcone. Chalcone isomerase converts tetrahydroxychalcone into naringenin. ANR and ANS are all involved in the synthesis of anthocyanins. The results showed that the expressions of 6 genes, including *TRINITY_DN28401_c0_g1* (*CHI*), *TRINITY_DN36537_c1_g1* (*HCT*), *TRINITY_DN37574_c1_g2* (*HCT*), *TRINITY_DN39063_c0_g1* (*FLS*), *TRINITY_DN43344_c1_g1* (*CHI*) and *TRINITY_DN41734_c0_g5* (*CHS*), were significantly upregulated, and 4 genes, including *TRINITY_DN42700_c3_g2* (*F3H*), *TRINITY_DN43120_c5_g1* (*ANR*), *TRINITY_DN44094_c0_g1* (*ANS*) and *TRINITY_DN44565_c1_g1* (*F3M*), were significantly downregulated. (Fig. 4) From the integrated metabolic map, it could be seen that the expressions of upstream genes in the phenylpropane metabolic pathway were increased with MeJA treatment. And the results of gene expression also suggested that these genes might be involved in metabolic pathways.

### Elements analysis of the promoters differentially expressed flavonoid biosynthesis genes

**Fig. 5:**
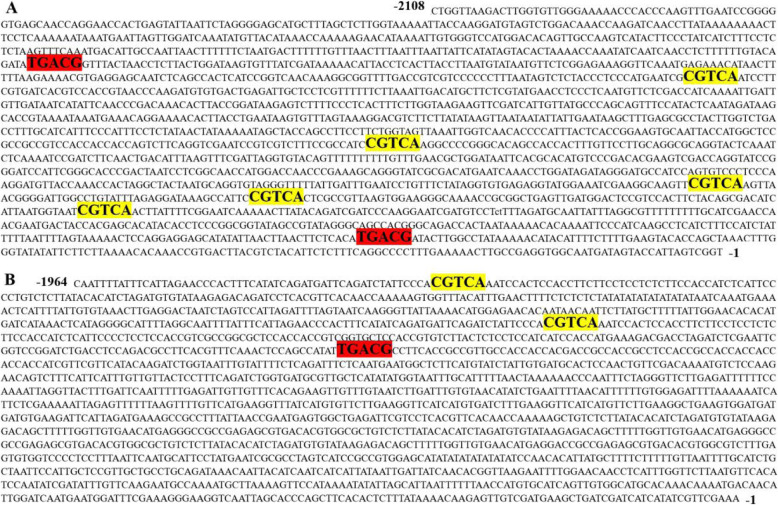
Identification of MeJA response elements found in the promoter sequences of *pCtCHI* (**a**) and *pCtHCT* (**b**). A is the sequence analysis for *pCtCHI.* B is the sequence analysis for *pCtHCT.* The sequences were analyzed by PlantCARE (http://bioinformatics.psb.ugent.be/webtools/plantCARE). The elements in the yellow indicated that the sequence of MeJA responded element was the same as it (sense strand of DNA), while the elements in the red was that as the reverse complementary sequence (antisense strand of DNA)

To analyse how MeJA regulates gene expression, the promoters of differentially expressed flavonoid biosynthesis genes were cloned and analysed. Because there were no reference genome sequences from safflower, single oligonucleotide nested PCR (SON-PCR) was used to clone the promoters [27]. In our study, two promoters of MeJA significantly upregulated genes *CtCHI* and *CtHCT* (named *pCtCHI* and *pCtHCT,* respectively) were successfully cloned, and the fragments were cloned into the T vector and transformed into *DH5a* bacteria. A positive bacterial solution was selected for sequencing. The sequences were shown in Fig. 5.

The sequences of the promoters were analysed using PlantCARE (http://bioinformatics.psb.ugent.be/webtools/plantCARE) [28]. The sequences upstream of ATG were submitted to a web analysis program. Seven MeJA response elements could be found in the promoters of *pCtCHI*, while three MeJA response elements could be found in the promoters of *pCtHCT.* (Fig. 5) The results implied that MeJA could regulate these gene expressions through these elements, thus promoting the synthesis of quinone chalcones.

## Discussion

Many secondary metabolites can be induced by MeJA, such as volatile, stilbene, carotenoids, unsaturated fatty acids, flavonoids, lycopene, among others [29–31]. Flavonoids are one of the most reported compounds among them. However, not all the results showed that flavonoids biosynthesis can be upregulated by MeJA. Farag made the analysis of *Erythrina lysistemon* cell suspension culture in response to MeJA elicitation, and results revealed that triterpene i.e. oleanolic acid and fatty acid i.e. hydroxy-octadecadienoic acid were elicited in response to MeJA, whereas pterocarpans i.e. isoneorautenol showed a decline in response to MeJA elicitation [32]. Our results showed that 24 components of 35 differentially metabolized flavonoids could be induced and 11 components were reduced.

It have been reported that 104 compounds from safflower were isolated and detected [33]. In previous research [13], 12 flavonoid compounds were detected, including rutin, HSYA, kaempferol-3-O-β-D-rutinoside, kaempferol-3-O-β-D-glucoside, carthamin, luteolin, and quercetin-3-O-β-D-glucoside. We found that rutin, HSYA, kaempferol-3-O-β-D-rutinoside, carthamin, and quercetin-3-O-β-D-glucoside were induced by MeJA treatment. In our results, 209 flavonoid compounds were detected by UHPLC-ESI-MS/MS with a self-built database. We profiled the chemical compositions of safflower in a systematic and comprehensive way, which provided a reference for their sufficient utilization in the future. UHPLC-ESI-MS/MS was an effective method to thoroughly understand plant secondary metabolites.

In previous reports of MeJA regulating flavonoids biosynthesis, many studies focused on the analysis of the genes expression. In *Arabidopsis*, MeJA similarly induced expression of almost all anthocyanin biosynthetic genes (*PAL*, *CHS*, *CHI*, *F3H*, *F3’H*, *DFR* and *UFGT*), but *PAL*, *CHS*, *CHI* and *F3H* were at only low levels. Further analysis show that the late anthocyanin biosynthesis genes (such as *DFR* and *UFGT*), were found to be up-regulated strongly by MeJA [34]. While in *Gynura bicolor* DC., the expression of flavonoid biosynthesis genes, *GbCHS*, *GbCHI*, *GbDFR* and *GbANS*, was markedly up-regulated. Compared with that in *Arabidopsis*, the genes, which are classified as up flavonoid biosynthesis genes, were found to be up-regulated strongly by MeJA [35]. From the studies above, it can be indicated that the flavonoid biosynthesis genes expression varied among different plants in response to the MeJA treatment. In our study, MeJA could upregulate the expression of upstream genes in the flavonoid biosynthesis pathway (*CHSs*, *CHIs*, and *HCTs*) and downregulate the expression of downstream genes (*F3Ms*, *ANRs*, and *ANSs*), thus promoting the biosynthesis of quinone chalcones, such as HSYA. It is probably that MeJA could upregulate the expression of *TRINITY_DN28401_c0_g1* (*CHI*), *TRINITY_DN36537_c1_g1* (*HCT*), *TRINITY_DN37574_c1_g2* (*HCT*), *TRINITY_DN43344_c1_g1* (*CHI*), and *TRINITY_DN41734_c0_g5* (*CHS*), and downregulate the expression of *TRINITY_DN42700_c3_g2* (*F3H*), *TRINITY_DN43120_c5_g1* (*ANR*), *TRINITY_DN44094_c0_g1* (*ANS*), and *TRINITY_DN44565_c1_g1* (*F3M*), thus promoting the biosynthesis of quinone chalcones (Fig. 4). Of course, to make more conclusive evidence, some other experiments, such as enzyme activity analysis and transgenic experiments, are required.

In the research of *Gynura bicolor* DC., some genes were markedly up-regulated under MeJA treatment, such as *GbCHS*, which is expressed more than 100-fold as compared with that before MJ treatment [35]. However, there was no such high change in our results. The main reason might be the different way of dealing with MeJA. In previous research, MeJA was added into 20 ml fresh MS liquid medium, where *Gynura bicolor* DC. was planted, while in our research, MeJA was sprayed onto healthy safflower flowers. The amounts for plant exposed to MeJA were different.

Since there is no reference genome in safflower, it is still difficult to clone the fragment. However, we fortunately cloned two promoters of up-regulated gene (*HCT* and *CHI*) in our experiment. The sequence analysis results showed that there were MeJA response elements on the promoters, which further proved the RT-PCR results. Athough our results showed that MeJA upregulate the expression of upstream genes in the flavonoid biosynthesis pathway and downregulate the expression of downstream genes, thus promoting the biosynthesis of quinone chalcones, such as HSYA, what genes MeJA regulated to promote the production of HSYA is still unknown, as the genes involved in the biosynthesis of HSYA have not been fully identified. Future researches can be strengthened in this domain.

## Conclusions

Here, we used metabolomics and transcriptome technologies to analyse the molecular mechanism of flavonoid biosynthesis under MeJA treatment in safflower. Based on a UHPLC-ESI-MS/MS detection platform and a self-built database (including HSYA), a total of 209 flavonoid metabolites were detected, and 35 metabolites were significantly different. Among them, 24 metabolites were upregulated upon MeJA treatment, especially HSYA. Eleven metabolites were downregulated after MeJA treatment. Integrated metabolomics and transcriptome analysis showed that MeJA might upregulate the expression of upstream genes in the flavonoid biosynthesis pathway (such as *CHSs*, *CHIs* and *HCTs*) and downregulate the expression of downstream genes (such as *F3Ms*, *ANRs* and *ANSs*), thus promoting the biosynthesis of quinone chalcones, such as HSYA. The transcription expressions of these genes were validated by real-time PCR. In addition, the promoters of two genes that were significantly upregulated under MeJA treatment were cloned and analysed. Ten MeJA response elements were found in the promoters. Our results provide insights and basic data for the molecular mechanism analysis of flavonoid synthesis in safflower under MeJA treatment.

## Methods

### Plant materials

**Fig. 6:**
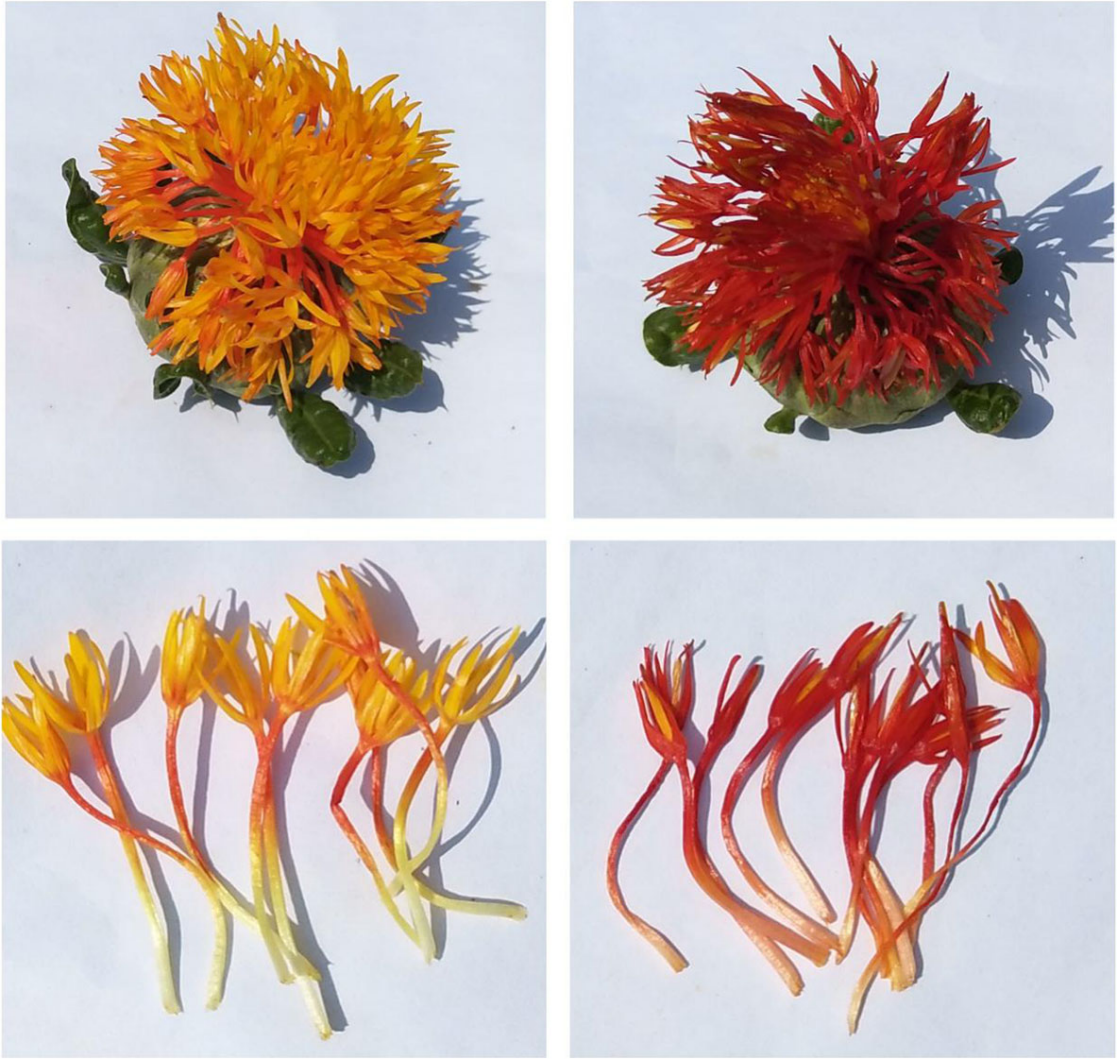
The flowers of safflower treated with or without MeJA. **a** The inflorescences of safflower treated without MeJA (CK). **b** The inflorescences of safflower treated with MeJA. **c** The tubular flowers of safflower treated without MeJA (CK). D The tubular flowers of safflower treated with MeJA. The bar was 1 cm

Safflower used in this experiment was named as “Chuanhonghua No.1”, which is cultivated by Industrial Crop Research Institute, Sichuan Academy of Agricultural Sciences. It was presented by Renchuan Yao and identified as safflower (*Carthamus tinctorius* L.) by professor Pei Jin. It was cultivated at the medicinal botanical garden on the Wenjiang Campus of Chengdu University of Traditional Chinese Medicine. The treatment was primarily applied according to the previous report with some modifications [13]. A 100 μM solution of MeJA (Sigma-Aldrich, Switzerland) was sprayed onto healthy safflower flowers 3 days after anthesis (DAA). In the control group, the flowers were sprayed with the same solution but without MeJA. The flowers were then enclosed in clear plastic bags to prevent the emission of volatile phytohormones and to allow for the elicit or solutions to be more highly absorbed. After treatment for 6 h, the plastic bags were removed, and samples of flowers were collected, frozen immediately in liquid nitrogen and stored in a freezer at − 80 °C. For the RNA sequencing, five inflorescences of safflower were mixed as a sample. As the RNA sequencing results were verified by the real-time PCR experiments (the experimental method was listed below) in the study, there were no replicates for the RNA sequencing. For metabolism analysis, ten inflorescences of safflower plants were mixed as one sample. Three biological replicates were performed for the metabolomics analysis. The flowers of safflower with or without MeJA treatment are shown in Fig. 6.

### Sample preparation and extraction for metabolomic analysis

The freeze-dried flowers were crushed using a mixer mill (MM 400, Retsch) with a zirconia bead for 1.5 min at 30 Hz. One hundred milligrams of powder was weighed and extracted overnight at 4 °C with 1.0 mL 70% aqueous methanol. Following centrifugation at 10000 *g* for 10 min, the extracts were absorbed (CNWBOND Carbon-GCB SPE Cartridge, 250 mg, 3 mL; ANPEL, Shanghai, China, www.anpel.com.cn/cnw) and filtered (SCAA-104, 0.22 μm pore size; ANPEL, before LC-MS analysis.

### UHPLC conditions

The sample extracts were analysed using an LC-ESI-MS/MS system (HPLC, Shim-pack UFLC SHIMADZU CBM30A system; MS, Applied Biosystems 6500 Q TRAP). The analytical conditions were as follows: UHPLC column, Waters ACQUITY UHPLC HSS T3 C18 (1.8 μm, 2.1 mm × 100 mm); solvent system, A water (0.04% acetic acid): B acetonitrile (0.04% acetic acid); gradient program, 0% B at 0 min, 95% B at 11.0 min, 95% B at 12.0 min,5% B at 12.1 min, and 5% B at 15.0 min; flow rate, 0.40 mL/min; temperature, 40 °C; and injection volume, 2 μL. The UHPLC effluent was connected to an ESI-triple quadrupole-linear ion trap (Q TRAP)-MS.

### ESI-q trap-MS/MS

Mass spectrometry followed the method of Chen et al. [19]. LIT and triple quadrupole (QQQ) scans were acquired on a triple quadrupole-linear ion trap mass spectrometer (Q TRAP), API 6500 QTRAP LC/MS/MS system equipped with an ESI Turbo ion-spray interface, operating in positive ion mode and controlled by Analyst 1.6.3 software (AB Sciex, Waltham, MA, USA). The ESI source operation parameters were as follows: ion source, turbo spray; source temperature, 500 °C; ion spray voltage (IS), 5500 V; the ion source gas I (GSI), gas II (GSII), and curtain gas (CUR) were set at 55, 60, and 25.0 psi, respectively; and the collision gas (CAD) was high. Instrument tuning and mass calibration were performed with 10 and 100 μmol/L polypropylene glycol solutions in QQQ modes. QQQ scans were acquired as MRM experiments with collision gas (nitrogen) set to 5 psi. DP and CE for individual MRM transitions was performed with further DP and CE optimization. A specific set of MRM transitions was monitored for each time period according to the metabolites eluted within that period.

### Qualitative and quantitative analysis of metabolites

The flavonoid identification and quantification in our study was made according to a method of scheduled multiple reaction monitoring (MRM), which has been previously described [19]. With this methods, Chen made a genome-wide association analyses between genetic and biochemical, and reported 840 metabolites, from which 277 were identified or annotated [20]. Mainly based on this research, a database was built. All the metabolites in our study were detected or annotated by this database. The database and methods have been wildly used to detected or annotate the metabolites, such as the research of Wang [36] and Liu [37]. This methods is also called as wild-targetd analysis. As HSYA was not in the database, we use its standard sample (No.: MUST-15072815, Chengdu Manster Biotechnology Co., LTD, China) to build a library and add it to the self-built database. The metabolites of the samples were qualitatively and quantitatively analysed by mass spectrometry. The characteristic ions of each substance were filtered by the triple quadrupole, and the signal strength of the characteristic ions was obtained in the detector. Chromatographic peaks were analyzed with Multi Quant software 3.0.3. The integrated area peak of each compound was used for the PCA and OPLS-DA analysis..

### Differential metabolite analysis

Principal component analysis (PCA) can effectively extract main variance information and was used in many other research [29, 38]. In addition, orthogonal partial least squares-discriminant analysis (OPLS-DA) was used for variables with less correlation. The experiment had biological duplication; thus, the fold change and VIP value of the OPLS-DA model were combined to screen differential metabolites. PCA was analyzed by R software built-in functions (www.r-project.org/). The parameter: scale = True. After conversion of the original data by log2, the data was centralized (Mean Centering) and analyzed by OPLSR. Anal of Metabo Analyst in R software. The main steps of our study can be referred to a previouly research [39]. Screening criteria: 1. Metabolites must exhibit fold changes≥2 and fold changes≤0.5. If a metabolite in the experimental groupwas more than 2 times or less than 0.5 times that of the metabolite in the control group, the difference was considered significant. 2. If there is biological duplication in the sample grouping, the metabolites of VIP ≥1 are selected on the basis of the above. The VIP value indicates the influence intensity of the difference between groups of corresponding metabolites in the classification and discrimination of each group of samples in the model.

### RNA sequencing and annotation

RNA isolation and purification and cDNA library construction and sequencing were performed as previously described [40]. All tissues were ground on dry ice, and total RNA was prepared by using TRIzol reagent (Invitrogen, CA, USA). To remove DNA, an aliquot of total RNA was treated with DNase (Takara, Dalian, China). RNA quantity and quality were determined by using a NanoDrop 2000 spectrophotometer (NanoDrop Technologies, Wilmington, DE, USA) and an Agilent 2100 Bioanalyser (Agilent Technologies, CA, USA), respectively. mRNA was isolated from total RNA using magnetic beads with oligo (dT) primer; cDNA was synthesized using a cDNA synthesis kit (TaKaRa, Dalian, China) and linking the sequencing adapter to both ends. The library preparations were sequenced on an Illumina HiSeq 4000 platform, and the unigene sequences obtained from our laboratory transcriptome database by RSEM (RNAseq by Expectation-Maximization) software were integrated for annotation. The whole set of transcript data can be found in the National Center for Biotechnology Information (NCBI) SRA database (SRR10011980 and SRR10011979).

### Screening of differential genes

DESeq2 [41, 42] was used for differential expression analysis among sample groups. DESeq2 requires the input of unregulated read counting data of genes rather than RPKM (Reads Per Kilobase Million), FPKM (Fragments Per Kilobase Million) and other standardized data. Read counts of genes are the expected_count outputs calculated using RSEM (RNAseq by Expectation-Maximization). The expected count is generally lower than the read number. After discrepancy analysis, multiple hypothesis tests are needed to correct the hypothesis test probability (*P* value) with the Benjamini-Hochberg procedure to obtain the false discovery rate (FDR) when |log_2_fold change| ≥ 1and FDR < 0.05.

### Real-time PCR analysis

Real-time PCR analysis was performed as previously described [14]. Total RNA was isolated using an RNA extraction kit (Invitrogen, CA, USA), and reverse transcription was carried out using the prime script reagent kit (Takara, Dalian, China). The primers used to amplify the screened genes (*CHS*, *CHI*, *HCT*, etc.) by real-time PCR were designed by Primer 5.0, and parts of the safflower *28S* coding region were used as an internal reference gene. The primer details are listed in Supplementary Table 4. All of the primers were tested for their specificity by agarose gel electrophoresis. RT-PCR was performed using an SYBR prime script RT-PCR kit (Takara) with three replicates, and the cycling conditions were set according to the manual. The Bio-Rad CFX96 real-time PCR detection system (Hercules, CA, USA) was used in our experiment.

### Promoter cloning and sequence analysis

Because there is no complete genome sequence for safflower, the 5′ flanking region of the flavonoid gene was isolated from genomic DNA of the safflower plant using SON-PCR. The reactions (50 μL) were performed with 200 μmol of each dNTP, 2 μmol of primer and 2 units of LA Taq DNA polymerase (Takara). The primer details are listed in Supplementary Table 4. The resultant PCR fragment, measuring approximately 2 kb, was cloned into a *pMD19-T* vector (Takara) and sequenced. The flavonoid gene promoter sequence was analysed with the PLACE Web SignalScan program (http://bioinformatics.psb. ugent.be/webtools/plantCARE [28];.

## Supplementary information

**Additional file 1: Table S1.** List of the 209 metabolites detected in safflower samples and OPLS-DA results. Two hundred nine flavonoid metabolites details were listed in the table. CK represent treatment without MeJA, M-MeJA represent treatment with MeJA. Three biological repeats were made in the experiment. **Table S2.** Extract of the 35 metabolites significantly different between MeJA and non-treated materials. CK represents treatment without MeJA. Three biological replicates were used in the experiment. **Table S3.** Details of the transcripts belonging to the flavonoid biosynthesis which were significantly different after MeJa treatment. M-CK represent treatment without MeJA, M-MeJA represent treatment with MeJA. **Table S4.** List of primers used for RT-PCR and promoter cloning experiments.

## Data Availability

The raw data was uploaded to Sequence Read Archive (SRR10011980 and SRR10011979).

## Abbreviations

CHI: Chalcone isomerase
CHS: Chalcone synthase
F3H: Flavonoid 3-hydroxylase
UGT: UDP-glucuronosyl transferases
HCT: Hydroxyl-cinnamoyltransferase
F3Ms: Flavonoid 3′-monooxygenase
ANRs: Anthocyanidin reductase
ANSs: Anthocyanidin synthase
MeJA: Methyl jasmonate
KEGG: Kyoto Encyclopedia of Genes and Genomes
HSYA: Hydroxysafflor yellow A
CK: Control check
MeJA: Methyl jasmonate
